# Dr. A. W. Griggs

**Published:** 1890-06

**Authors:** 


### DR. A. W. GRIGGS.

The election of Dr. Griggs to the Presidency of the Medical Association of Georgia is a well-deserved compliment. Than Dr. Griggs there is no better or more successful practitioner in the State, and no one could have been selected to fill that important position who is more eminently fitted for the place. With his mind so well stored by long experience, close observation and wide reading with valuable medical facts; with his facetious style of oratory and his exhaustless fund of anecdotes, there is a treat in store for those who attend the next meeting of the Association.

